# Fruit and vegetable consumption in relation to ovarian cancer incidence: the Swedish mammography cohort

**DOI:** 10.1038/sj.bjc.6601872

**Published:** 2004-05-04

**Authors:** S C Larsson, L Holmberg, A Wolk

**Affiliations:** 1 Division of Nutritional Epidemiology, The National Institute of Environmental Medicine, Karolinska Institute, Box 210, SE-171 77 Stockholm, Sweden; 2 Regional Oncologic Center, University Hospital, SE-75185 Uppsala, Sweden

**Keywords:** cohort studies, diet, epidemiology, fruits, ovarian cancer, vegetables

## Abstract

We prospectively examined the incidence of epithelial ovarian cancer and its subtypes in relation to baseline fruit and vegetable consumption in the Swedish Mammography Cohort, a population-based cohort study of 61 084 women aged 38–76 years in 1987–1990. During an average follow-up of 13.5 years, 266 incident cases of invasive epithelial ovarian cancer were diagnosed. After adjustment for potential confounders, we observed a statistically significant inverse association between consumption of vegetables and ovarian cancer risk (*P-*value for trend=0.01); the multivariate rate ratio (RR) for the comparison of three or more servings of vegetables per day with one or fewer servings per day was 0.61 (95% confidence interval (CI), 0.38–0.97). For fruit consumption a modest, not statistically significant, positive association was found (*P*-value for trend=0.07); the multivariate RR for the highest compared with the lowest category of consumption being 1.37 (95% CI, 0.90–2.06). The associations with fruit and vegetable consumption did not vary by subtype of ovarian cancer. These findings suggest that high consumption of vegetables, but not of fruits, may reduce the risk of ovarian cancer.

As for many other cancers, high consumption of fruit and vegetables is supposed to reduce the risk of ovarian cancer. In addition to being a major source of dietary vitamin C, folate, and fibre, fruits and vegetables also contain numerous other potentially anticarcinogenic phytochemicals (Park and Pezzuto, 2002). The majority of case–control studies (La Vecchia *et al*, 1987; Engle *et al*, 1991; Bertone *et al*, 2001; Bosetti *et al*, 2001; Cramer *et al*, 2001; McCann *et al*, 2001; Zhang *et al*, 2002; McCann *et al*, 2003), although not all (Shu *et al*, 1989; Salazar-Martinez *et al*, 2002), have suggested an inverse association of consumption of total vegetables or of certain subgroups of vegetables with ovarian cancer risk. In two prospective cohort studies that have reported results for consumption of total vegetables and ovarian cancer risk (Kushi *et al*, 1999; Fairfield *et al*, 2001), a nonsignificant approximately 25% reduction in ovarian cancer risk was observed for the highest in comparison with the lowest category of consumption. A recent comprehensive review of the literature on fruit and vegetable consumption and cancer by the IARC Working Group on the Evaluation of Cancer-Preventive Strategies (2003) concluded that ‘an increase in consumption of vegetables possibly reduces the risk of ovarian cancer’. In contrast to vegetable consumption, fruit consumption in case–control (Shu *et al*, 1989; Bosetti *et al*, 2001; McCann *et al*, 2001,^2003^; Salazar-Martinez *et al*, 2002; Zhang *et al*, 2002) and cohort studies (Kushi *et al*, 1999; Fairfield *et al*, 2001) of ovarian cancer have yielded conflicting results, with both inverse and positive associations.

We have therefore examined overall fruit and vegetable consumption as well as consumption of specific fruits and vegetables in relation to total ovarian cancer incidence and its subtypes in a large prospective population-based cohort of Swedish women.

## SUBJECTS AND METHODS

### Study population

The Swedish Mammography Cohort is an ongoing population-based prospective study of 66 651 women who were 38–76 years of age and living in Uppsala and Västmanland counties in central Sweden when they responded to a mailed questionnaire in 1987–1990. The questionnaire sought information on diet, parity, age at first birth, family history of breast cancer, education level, weight, and height. Data on age at menarche, age at menopause, and use of exogenous hormones were received only from women in Uppsala County at the time of their mammography examination. In 1997, all surviving participants were mailed a follow-up questionnaire that inquired about age at menarche, age at menopause, and history of oral contraceptive and postmenopausal hormone use. This study was approved by the Ethics Committees at the Uppsala University Hospital (Uppsala, Sweden) and the Karolinska Institutet (Stockholm, Sweden).

In the current analysis, we excluded women with inadequate dietary data (i.e. reported an energy intake that was three standard deviations below or above the mean value for log-transformed energy), women with a previously diagnosed cancer (other than nonmelanoma skin cancer). Furthermore, by linkage with the Swedish Inpatient Register, we identified and excluded all women who had a bilateral oophorectomy or a hysterectomy with unknown number of ovaries removed before baseline. After these exclusions, a total of 61 084 women remained for this analysis.

### The food-frequency questionnaire

The self-administered food-frequency questionnaire covered 67 food items over the past 6 months. Participants chose from among eight possible responses, ranging from never/seldom to four or more times per day. We used age-specific (<53, 53–65, ⩾66 years) portion sizes that were based on mean values obtained from 213 randomly chosen women from the study area whose food intake for 5922 days was weighed and recorded (A Wolk, unpublished data). Nutrient intakes were computed by multiplying the consumption frequency of each food item by the nutrient content per serving, using composition values obtained from the Swedish National Food Administration Database (Bergström *et al*, 1991). Total vegetable consumption included tomatoes, cucumber, iceberg lettuce, china cabbage, spinach, kale, cabbage, carrots, and beets; total fruit consumption included apples, pears, bananas, oranges, mandarins, and grapefruit (fruit juice was not included). Missing responses for vegetable and fruit items were considered as zero consumption.

In a study of the validity of the food-frequency questionnaire among 129 women randomly selected from the cohort, Pearson's correlation coefficients between the average of four 1-week diet records (obtained 3–4 months apart) and estimates from the questionnaire ranged from 0.4 to 0.6 for individual fruit items and from 0.2 to 0.5 for individual vegetable items.

### Identification of ovarian cancer cases and follow-up of the cohort

Incident cases of invasive epithelial ovarian cancer were identified by linkage of the study population with the national Swedish Cancer Registry (from March 1987 to 31 December 1997) and the Regional Cancer Registry in the study area (from 1 January 1998 to 30 June 2003). Follow-up of the cohort through these registries is estimated to be 98% complete (Mattsson and Wallgren, 1984). Deaths were ascertained through the Swedish Death Registry and information with regard to date of leaving the study area was obtained from the Swedish Population Registry.

### Statistical analysis

For each participant, person-years of follow-up accrued from the entry to the cohort (i.e. the date of mammography screening) and ended at the date of diagnosis of ovarian cancer, of a bilateral oophorectomy or a hysterectomy with unknown ovaries removed, of death, of migration out of the study area, or the end of the follow-up period (30 June 2003), whichever came first. Participants were grouped into categories of fruit and vegetable consumption, and age-specific incidence rates of ovarian cancer were calculated by dividing the number of incident cases by the number of person-years in each category. Rate ratios (RRs) of ovarian cancer were computed as the incidence rate in a specific category as compared with that for the reference category (i.e. the lowest consumption category). The data met the assumptions for using Cox's proportional-hazards modelling, and we used this method to estimate RRs (with 95% confidence intervals (CI)), controlled for age at baseline, body mass index, educational level, parity, use of oral contraceptives, fish consumption, and dietary lactose intake. We also assessed the potential confounding by age at menarche, age at first birth, age at menopause, postmenopausal hormone use, and family history of breast cancer. However, as adjustment for these variables did not appreciably alter the results, we omitted them from the final multivariate model. We performed tests for trend across categories by assigning the median value to each category and modelling this variable as a continuous variable. All reported *P*-values are based on two-sided tests.

## RESULTS

Among 61 084 women followed for an average of 13.5 years (823 572 person-years), a total of 266 women were diagnosed with invasive epithelial ovarian cancer, including 125 serous cancers, 48 endometrioid cancers, and 21 mucinous cancers; 72 cancers were of other histologic subtypes or could not be classified. Baseline characteristics of the cohort by categories of consumption of total fruit and total vegetables are shown in Table 1

**Table 1:** Age-standardised baseline characteristics according to the consumption of total vegetables and total fruits in the Swedish Mammography Cohort

	**Vegetable consumption**		**Fruit consumption**	
	**Lowest category**	**Highest category**	**Lowest category**	**Highest category**
Mean age at baseline (years)	54.9	53.3	53.4	53.9
Mean body mass index (kg/m^2^)	24.8	25.0	24.7	24.9
Mean no. of children	2.1	2.1	2.1	2.1
Education ⩾12 years (%)	10.0	14.9	11.6	13.4
Ever used oral contraceptives (%)	54.1	52.5	53.4	52.6
Mean dietary intake				
Lactose^a^ (g/day)	12.8	11.7	12.7	10.6
Fish (servings/week)	0.5	0.9	0.5	0.8

a Adjusted for total energy intake by regression analysis (Willett and Stampfer, 1986).

. Compared with women in the lowest categories of consumption, those in the highest categories were more educated and had a higher intake of fish and a lower intake of lactose. Women in the highest category of vegetable consumption also had a lower prevalence of oral contraceptive use compared with those in the lowest category.

Overall fruit and vegetable consumption was not appreciably associated with ovarian cancer risk after adjusting for potential confounders. Women who consumed five or more servings per day of fruit and vegetables combined had a multivariate RR for ovarian cancer of 0.78 (95% CI, 0.51–1.20) compared with those who consumed less than two servings per day (data not shown). When we examined total vegetables and total fruits separately, a significant inverse association was observed between consumption of vegetables and risk; the multivariate RR for the highest (⩾3 servings day^−1^) compared with the lowest (⩽1 serving day^−1^) category of consumption was 0.61 (95% CI, 0.38–0.97) (Table 2

**Table 2:** RR and 95% CI of invasive epithelial ovarian cancer by consumption of total vegetables and total fruits

	**Category of consumption, servings/day**				
	**⩽1.0**	**1.1 to <2.0**	**2.0 to <3.0**	**⩾3.0**	***P-*value for trend**
*Total vegetable consumption*					
Cases/person-years	105/283 419	89/272 804	48/171 844	24/95 505	
Age-adjusted RR (95% CI)	1.00	0.93 (0.70–1.23)	0.79 (0.56–1.11)	0.71 (0.46–1.11)	0.07
Multivariate^a^ RR (95% CI)	1.00	0.87 (0.65–1.16)	0.70 (0.49–1.00)	0.61 (0.38–0.97)	0.01
Total fruit consumption					
Cases/person-years	111/282 300	66/299 952	58/155 070	31/86 250	
Age-adjusted RR (95% CI)	1.00	0.87 (0.63–1.21)	1.07 (0.79–1.46)	1.07 (0.74–1.56)	0.47
Multivariate^a^ RR (95% CI)	1.00	1.14 (0.84–1.55)	1.31 (0.95–1.82)	1.37 (0.90–2.06)	0.07

a Multivariate RRs were adjusted for age at baseline (in 5-year categories), body mass index (in quartiles), educational level (less than high school, high school, university), parity (nulliparous, 1–2, ⩾3 children), oral contraceptive use (ever or never), fish consumption (in quartiles), and dietary lactose intake (in quartiles). Consumption of total fruit and total vegetables were mutually adjusted. Total vegetables included tomatoes, cucumber, iceberg lettuce, china cabbage, spinach, kale, cabbage, carrots, and beets; total fruits included apples, pears, bananas, and citrus fruit (oranges, mandarins, grapefruit). RR=rate ratios; CI=confidence intervals.

). Assessed as a continuous trend, an increment of one serving of vegetables per day corresponded to a 10% decrease in risk of ovarian cancer (multivariate RR, 0.90; 95% CI, 0.80–1.01). Although not statistically significant, a weak positive association was observed with the consumption of total fruits in a multivariate model that adjusted for vegetable consumption and other potential confounders (RR, 1.37; 95% CI, 0.90–2.06, for the highest *vs* the lowest category) (Table 2). Adding fruit juice to total fruit consumption yielded similar results (multivariate RR, 1.33; 95% CI, 0.91–1.95).

We next examined the possible associations of individual vegetable and fruit items with ovarian cancer risk (Table 3

**Table 3:** RR and 95% CI of invasive epithelial ovarian cancer by consumption of specific vegetables and fruits

	**Category of consumption**				
	**1**	**2**	**3**	**4**	***P-*value for trend**
*Cabbage*					
Servings/week	0	0.1 to <0.5	0.5 to <1.5	⩾1.5	
Cases/person-years	73/197 160	109/335 301	42/138 423	42/152 688	
Multivariate^a^ RR (95% CI)	1.00	0.91 (0.67–1.25)	0.88 (0.59–1.32)	0.87 (0.58–1.31)	0.49
					
*Carrots and beets*					
Servings/week	<1.0	1.0 to <3.0	3.0 to <5.0	⩾5.0	
Cases/person-years	50/147 686	60/171 983	76/258 319	80/245 584	
Multivariate^a^ RR (95% CI)	1.00	0.97 (0.66–1.43)	0.79 (0.54–1.15)	0.86 (0.58–1.28)	0.55
					
*Green leafy vegetables*^b^					
Servings/week	<1.5	1.5 to <3.0	3.0 to <7.0	⩾7.0	
					
Cases/person-years	81/220 252	77/227 851	52/175 542	56/199 927	
Multivariate^a^ RR (95% CI)	1.00	1.00 (0.71–1.40)	0.83 (0.55–1.25)	0.83 (0.55–1.25)	0.22
					
*Tomatoes*					
Servings/week	<1.0	1.0 to <3.0	3.0 to <5.0	⩾5.0	
No. of cases	85/238 278	80/251 869	50/149 462	51/183 963	
Multivariate RR	1.00	0.99 (0.71–1.38)	1.18 (0.79–1.75)	0.86 (0.57–1.30)	0.74
					
*Apples and pears*					
Servings/week	<1.5	1.5 to <3.0	3.0 to <7.0	⩾7.0	
Cases/person-years	73/239 437	55/179 297	29/104 056	114/300 782	
Multivariate^a^ RR (95% CI)	1.00	0.96 (0.66–1.40)	0.96 (0.61–1.51)	1.22 (0.89–1.69)	0.21
					
*Bananas*					
Servings/week	0	0.1 to <0.5	0.5 to <2.5	⩾2.5	
Cases/person-years	48/139 846	55/161 167	103/349 009	60/173 550	
Multivariate^a^ RR (95% CI)	1.00	1.06 (0.72–1.58)	0.96 (0.67–1.37)	1.10 (0.74–1.63)	0.67
					
					
*Citrus fruit*					
Servings/week	<1.5	1.5 to <3.0	3.0 to <7.0	⩾7.0	
Cases/person-years	69/216 471	36/114 657	84/280 465	77/211 979	
Multivariate^a^ RR (95% CI)	1.00	1.09 (0.72–1.64)	1.06 (0.75–1.50)	1.11 (0.78–1.59)	0.57

a Multivariate RRs were adjusted for the same variables as the multivariate model in Table 2.

b Green leafy vegetables included iceberg lettuce, china cabbage, spinach, and kale.

^c^Citrus fruit included oranges, mandarins, and grapefruit. RR=rate ratios; CI=confidence intervals.

). Although none of the individual vegetables or subgroups of vegetables – including cabbage, carrots and beets, green leafy vegetables, and tomatoes – alone accounted for the inverse association, the majority of the associations were weakly inverse. There was no important association between any individual fruit items and ovarian cancer risk.

The association of consumption of fruits and vegetables with risk was similar for serous (*P*-value for trend: fruit, 0.14; vegetables, 0.11) and for nonserous (*P*-value for trend: fruit, 0.27; vegetables, 0.06) histologic subtypes of ovarian cancer, and were consistent across subgroups defined by age, body mass index, parity, and use of oral contraceptives (data not shown). The results were essentially unaltered after omitting cases diagnosed during the first 2 years of follow-up (data not shown).

## DISCUSSION

In this large population-based prospective cohort study, we found no association between overall fruit and vegetable consumption and ovarian cancer risk. Nonetheless, a high consumption of vegetables was associated with a significantly lower ovarian cancer incidence, even after controlling for potential risk factors. For each daily serving increase in vegetable consumption, the risk decreased by 10%. We found no evidence for a reduction in ovarian cancer risk associated with high fruit consumption and, if any, the evidence was for a positive rather than an inverse association.

Only two previous prospective cohort studies – the Iowa Women's Health Study (Kushi *et al*, 1999) and the Nurses' Health Study (Fairfield *et al*, 2001) – have investigated the association between consumption of fruit and vegetables and ovarian cancer. In both studies, consumption of total vegetables was inversely associated with ovarian cancer risk, but the results were not statistically significant. The Iowa Women's Health Study (Kushi *et al*, 1999), however, observed a statistically significant 56% reduction associated with frequent consumption of green leafy vegetables. The association between total fruit consumption and ovarian cancer risk in the two cohort studies (Kushi *et al*, 1999; Fairfield *et al*, 2001) was null or slightly positive.

All four case–control studies on total vegetable consumption and ovarian cancer risk have reported an inverse association (Bosetti *et al*, 2001; McCann *et al*, 2001,^2003^; Zhang *et al*, 2002). Case–control studies on fruit consumption have yielded conflicting results. Although one study in China (Zhang *et al*, 2002) indicated a statistically significant inverse association between total fruit consumption and risk of ovarian cancer, four other studies found no association (Shu *et al*, 1989; Bosetti *et al*, 2001; McCann *et al*, 2001,^2003^) and one study in Mexico (Salazar-Martinez *et al*, 2002) reported a statistically significant positive association.

The strengths of this study are its population-based design, its large size, and the virtually complete case ascertainment through the Swedish Cancer Register system. In addition, the prospective nature of this study avoided recall and selection biases.

A potential limitation of any observational study is that dietary assessment is inevitably subject to measurement error. However, because our data were gathered before the diagnosis of ovarian cancer, misclassification would be expected to be nondifferential between cases and noncases and would tend to attenuate any true relationship. The somewhat limited number of fruit items listed on the food-frequency questionnaire probably resulted in some degree of measurement error, but this should not be related to disease status and only make our RR estimates conservative. Although we adjusted for a wide range of potential confounders, the possibility of unmeasured confounding or residual confounding cannot be entirely excluded.

In conclusion, findings from this population-based prospective cohort study suggest that a high consumption of vegetables, but not of fruits, may reduce the incidence of ovarian cancer. Our results along with a recent systematic review on fruit and vegetables by the IARC Working Group on the Evaluation of Cancer-Preventive Strategies (2003) provide support for the general public health recommendations to increase vegetable consumption.
